# Predictors of opioid efficacy in patients with chronic pain: A prospective multicenter observational cohort study

**DOI:** 10.1371/journal.pone.0171723

**Published:** 2017-02-03

**Authors:** Kasper Grosen, Anne E. Olesen, Mikkel Gram, Torsten Jonsson, Michael Kamp-Jensen, Trine Andresen, Christian Nielsen, Gorazd Pozlep, Mogens Pfeiffer-Jensen, Bart Morlion, Asbjørn M. Drewes

**Affiliations:** 1 Mech-Sense, Department of Gastroenterology & Hepatology, Aalborg University Hospital, Aalborg, Denmark; 2 Department of Clinical Medicine, Aarhus University, Aarhus, Denmark; 3 Department of Drug Design and Pharmacology, Faculty of Health and Medical Sciences, University of Copenhagen, Copenhagen, Denmark; 4 Department of Clinical Medicine, Aalborg University, Aalborg, Denmark; 5 Valdemar Hospital, Ringsted, Denmark; 6 Friklinikken, Region of Southern Denmark, Give, Denmark; 7 Department of Anaesthetics and Surgical Intensive Care, Ljubljana University Medical Centre, Ljubljana, Slovenia; 8 Department of Rheumatology, Aarhus University Hospital, Aarhus, Denmark; 9 Leuven Centre for Algology & Pain Management, University Hospitals Leuven, University Hospitals Leuven, Pellenberg, Belgium; Temple University, UNITED STATES

## Abstract

Opioids are increasingly used for treatment of chronic pain. However, they are only effective in a subset of patients and have multiple side effects. Thus, studies using biomarkers for response are highly warranted. The current study prospectively examined 63 opioid-naïve patients initiating opioid use for diverse types of chronic pain at five European centers. Quantitative sensory testing, electroencephalography (EEG) recordings, and assessment of pain catastrophizing were performed prior to treatment. The co-primary outcomes were change from baseline in ratings of chronic pain and quality of life after 14 days of opioid treatment. Secondary outcomes included patient’s global impression of clinical change and side effects. Logistic regression models adjusted for age and sex were used to identify biomarkers predictive for successful treatment, defined as at least a 30% reduction in average pain intensity or an improvement in quality of life of at least 10 scale points. Fifty-nine patients (94%) completed the study. The mean age was 55 ± 16 years and 69% were females. Pain reduction was predicted by cold pain intensity (OR: 0.69; *P* = 0.01), pain catastrophizing (OR: 0.82; *P* = 0.03), relative delta (OR: 0.76; *P* = 0.03) and beta EEG activity (OR: 1.18; *P* = 0.04) induced by experimental cold pain. None of the study variables were related to improvement in quality of life. For the first time, individual pain processing characteristics have been linked to opioid response in a mixed chronic pain population. This has the potential to personalize treatment of chronic pain and restrict opioid use to patients with high likelihood for response.

## Introduction

Chronic pain is a highly prevalent condition that may impact negatively on the individual’s quality of life; moreover, it is an expensive condition for society. Presently, chronic pain is considered as a bio-psychosocial phenomenon–a combination of physical dysfunction, beliefs, coping strategies, distress, illness behavior and social interactions. Pharmacotherapy is a cornerstone in the multimodal interdisciplinary treatment. However, chronic pain treatment still largely depends on local experience and traditions rather than individual patient characteristics [1, 2]. Chronic pain treatment, usually involving more than one drug, is seldom adjusted according to individual treatment response, but rather escalated or several therapies are tried in turn. Unfortunately, this strategy often leads to insufficient pain control, intolerable side effects, and psychosocial distress [3]. A deep concern has recently been raised about the uncritical prescription of opioids leading to growing abuse, addiction, and overdoses in the US [4]. Up to 50% of patients treated with morphine experience inadequate analgesia, despite escalating dose and/or experience intolerable or dose-limiting side effects [1]. This has driven a discovery-oriented research for biomarkers that can predict treatment response. However, due to the complexity of chronic pain, this approach has failed in clinical practice. Quantitative sensory testing, electroencephalography (EEG) recordings, and coping strategies have previously been used to identify patient subgroups in experimental studies and in highly selected chronic pain populations [5–8]. This includes morphine response in healthy volunteers, duloxetine efficacy in painful diabetic neuropathy, and pregabalin efficacy in painful chronic pancreatitis [9–11]. However, no studies have yet examined whether individual pain processing can predict efficacy of unselected opioids in a mixed patient population with chronic pain.

We hypothesized that the most promising techniques in previous experimental, postoperative, and clinical pain studies could serve as biomarkers of chronic pain and opioid analgesia. Hence, the objective of this study was to determine whether characteristics of altered pain processing in opioid-naïve patients could be used to predict clinically relevant treatment responses to opioids. To ensure general applicability, we collected longitudinal data from heterogeneous patient populations with moderate to severe chronic pain initiating opioid use at five centers in Europe.

## Methods

### Study design

The *Predictive Markers of the Effects of Opioid Therapy in Opioid-naïve Patients with Chronic Pain (ABILITY)* study was a two-week observational cohort study designed to evaluate the predictive value of pre-treatment markers of opioid response in opioid-naïve patients with various chronic pain conditions. This study was conducted across outpatient clinics at four university hospitals and one private hospital in Denmark, Slovenia, and Belgium. The research team recruited participants and conducted follow-up from October 2014 to September 2015. Opioid treatment of study patients took place without randomization and followed clinical practice at each participating center to reflect the true clinical situation. Opioid treatment was determined, prescribed, modified and discontinued at the sole discretion of the treating physician at each participating center; regardless of generic name, manufacturer, constituent components, route of administration, and dosing schedule (including titration and run-in periods). Regardless of any decision to modify or discontinue their assigned opioid treatment, study subjects were retained in the study whenever possible to enable follow-up data collection and prevent missing data. Of note, previously used pain medications were continued throughout the study. If opioid treatment was discontinued as a result of an adverse event, the circumstances leading to discontinuation of treatment were documented.

### Ethics statement and study registration

The Central Denmark Region Committees on Health Research Ethics (1-10-72-132-14) and the Danish Data Protection Agency (1-16-02-300-14/2007-58-0010) approved the study. The study protocol was further approved by the local ethics committees at each participating center. Participation in the study had no impact on the patients’ current or subsequent care and treatment. All study participants gave written informed consent. No financial compensation was given at any time during study. The study is registered at the US National Institutes of Health (ClinicalTrials.gov) #02308306.

### Data collection and follow-up

Patient demographics, medical history and medications, persistent pain features, quantitative sensory testing, EEG, and situational pain catastrophizing were registered and assessed at baseline. Data on clinical efficacy and side effects were collected at 14-days after initiating opioid treatment using a mailed questionnaire.

### Patients

The following main inclusion criteria were used: Pain duration ≥3 months; baseline pain intensity ≥4 and <9 on an 11-point numerical rating scale (NRS) ranging from 0 (*no pain*) to 10 (*worst pain imaginable*); expected to continue opioid and any concomitant non-opioid treatment throughout the study and age >18 years. Exclusion criteria were: Mental incapacity or language barriers precluding adequate understanding of study procedures; significant serious underlying conditions or similar reasons; having received opioid(s) on a daily basis (within the last 10 weeks); current alcohol and/or substance abuse.

### Outcome measures

The change from baseline in ratings of Brief Pain Inventory average pain scale (NRS 0–10) [12] and European Organization for Research and Treatment of Cancer Quality of Life questionnaire (QLQ-C30) health-related quality of life scores (0–100) [13] were chosen as co-primary efficacy endpoints in accordance with the Initiative on Methods, Measurement, and Pain Assessment in Clinical Trials (IMMPACT) recommendations [14, 15]. A clinically successful treatment response was defined as a pre-specified reduction in average pain intensity level above baseline, i.e., at least 30% (moderate to substantial improvement) [16, 17]. Similarly, a subjectively significant (moderate) change in quality of life scores was defined as ≥10 from baseline [18]. Secondary outcomes included change from baseline in ratings of the Brief Pain Inventory pain intensity items, patient-reported global impression of clinical change and adverse events.

### Predictors of clinically successful opioid treatment

#### Quantitative sensory testing

Electrical pain sensitivity was assessed on the volar forearm 2 cm distal to the wrist using a computer-controlled constant current stimulator (Isolator Stimulator Noxi IES 230, JNI Biomedical, Klarup, Denmark). The current intensity was gradually increased in steps of 1 mA until a sensation of pain was evoked (electrical pain detection threshold). Pressure pain sensitivity was assessed at the subjects’ quadriceps muscle 10 cm above the patella on the same side as the dominant hand using a handheld digital pressure algometer with a probe size of 1 cm^2^ (Algometer, Somedic AB, Hörby, Sweden). Pressure was gradually increased with a rate of 30 kPa per second until a pain sensation was evoked (pressure pain detection threshold). Cold pressor pain was induced by immersion of the patient’s hand in a stirred ice water bath (1–2°C) for two minutes. Patients were asked to rate worst cold pressor pain intensity (NRS 0–10) after two minutes of hand immersion (or upon spontaneous hand removal) and the pressure pain detection threshold was reassessed. The conditioned pain modulation effect was defined as the relative change in pressure pain thresholds before and after the cold pressor test. In brief, the ice water (conditioning stimulus) typically results in pain inhibition to the second pressure stimulus [19].

#### Electroencephalography recordings

Electrical activity of the brain was recorded during the two-minute cold pressor test via an electrode head cap with premeasured electrode placement (BIONEN Medical Devices, Firenze, Italy) using a digital amplifier (NuAmps, Neuroscan, El Paso, Texas, USA). Nine electrode sites and a reference electrode site were filled with Electro-Gel (Electro-Cap International, Eaton, OH, USA) and prepped to measured impedance values below 5 kohms. Pre-processing and spectral analysis of EEG dynamics have been described in detail elsewhere [10]. In brief, the absolute values of the obtained wavelet coefficients were used for analysis and divided into the following standardized frequency bands: Delta (1–4 Hz), theta (4–8 Hz), alpha (8–12 Hz) and beta (12–32 Hz). The wavelet coefficients were averaged over time and scales contained within each frequency band were summed together. The relative activity was then calculated separately for each channel by dividing each frequency band with the total energy of all bands and multiplying by 100; values then represented the percentage of total amplitude contained in each frequency band.

#### Situational pain catastrophizing

In connection with quantitative sensory testing, patients completed the Pain Catastrophizing Scale with reference to the pain induced by the cold pressor test [20]. Original instructions were appropriately revised [21]. Pain catastrophizing is characterized as the tendency to magnify the threat value of a pain stimulus and to feel helpless in the context of pain, and by a relative inability to inhibit pain-related thoughts in anticipation of, during or following a painful event [21].

### Study sample size

To date, no study has identified pre-treatment measures of opioid efficacy in opioid-naïve patients initiating opioid use for chronic pain and no a priori sample size could be calculated. The selection of clinically implementable measures for predicting opioid efficacy was therefore based on experiences from numerous clinical studies and by reviewing available literature [5]. We aimed at a sample size of 60 patients.

### Statistical methods

Analyses included all patients who took at least one dose of opioid medication and had at least one post-treatment efficacy assessment (complete case), irrespective of whether they continued opioid and any concomitant non-opioid treatment throughout the study or stopped treatment prematurely. Repeated outcome measurements and baseline characteristics between patients with and without clinically successful treatment were compared using the Wilcoxon signed-rank test and the Wilcoxon rank-sum test, respectively. Multilevel mixed-effects logistic regression models with a random intercept by participating center identification were applied to examine the influence of selected pre-treatment measures on opioid response. In brief, mixed effects logistic regression is used to model binary outcome variables when data are clustered or there are both fixed and random effects. In this study, a variety of outcomes were collected on patients, who were nested within clinics. By taking clustering within clinics into account, odds ratios (OR) and 95% confidence intervals (95% CI) were calculated with and without adjustment for sex and age. Concerning continuous predictors, it should be noted that the coefficient is the odds ratio for successful treatment per unit of measurement of difference in the explanatory variable. Two-tailed P-values <0.05 were considered statistically significant. Stata/IC 12.1 (StataCorp LP, College Station, TX, USA) was used for all statistical analyses.

## Results

**Fig 1:**
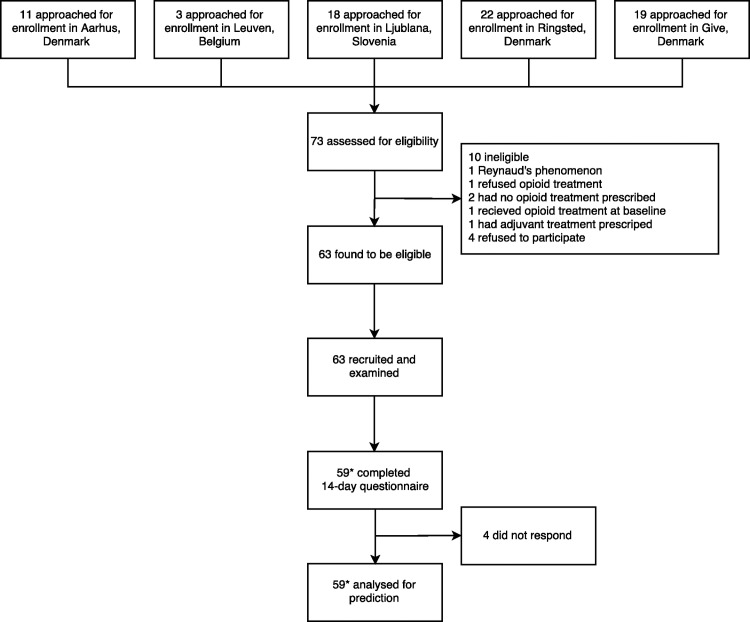
Flowchart. Numbers of patients screened for eligibility, examined, and analyzed for primary outcome.

The five participating centers screened 73 patients for eligibility (Fig 1). A total of 63 patients were enrolled and examined at baseline; 59 patients (94%) completed the post-treatment follow-up assessment. Demographic and baseline characteristics of patients are depicted in Table 1.

**Table 1:** Demographic and baseline characteristics of patients.

Clinical variables	
Participating center, number of participants	
Aarhus, DK	7 (12)
Leuven, BE	3 (5)
Ljubljana, SLO	17 (29)
Ringsted, DK	18 (31)
Give, DK	14 (24)
Sex (female/male)	41/18 (69/31)
Age, years	55 ±16
Weight, kg	79 ±17
Height, cm	171 ±10
BMI, kg/m^2^	27 ±5
Obese, BMI>30	15 (25)
Hand dominance (right/left)	56/3 (95/5)
Educational status	
Higher	6 (10)
Secondary	37 (63)
Basic	16 (27)
Employment status	
Employed	12 (20)
Unemployed	16 (27)
Retired	25 (42)
Other	6 (10)
Marital status (living with partner/alone)	47/11 (81/19)
Main site of pain	
Head, face, and mouth	2 (3)
Cervical region	6 (10)
Shoulder and upper limbs	14 (24)
Thoracic region	3 (5)
Lower back, lumbar spine, sacrum, coccyx	19 (32)
Lower limbs	7 (12)
More than three major sites	8 (14)
Abnormal system functioning producing pain	
Nervous system (CNS, PNS, autonomic)	28 (47)
Musculoskeletal system and connective tissue	30 (51)
More than one system	1 (2)
Temporal characteristics of pain	
Continuous, nonfluctuating	26 (44)
Continuous, fluctuating severity	31 (53)
Recurring irregularly	1 (2)
Recurring regularly	1 (2)
Patient's statement of pain intensity	
Medium—1 to 6 months	6 (10)
Medium—more than 6 months	15 (25)
Medium—1 month or less	1 (2)
Severe—1 to 6 months	5 (8)
Severe—more than 6 months	31 (53)
Unknown	1 (2)
Etiology	
Genetic or congenital disorders	3 (5)
Trauma, operation, burns	19 (32)
Infective, parasitic	1 (2)
Inflammatory	4 (7)
Neoplasm	1 (2)
Degenerative, mechanical	23 (39)
Unknown or other	8 (14)
Non-opioid analgesic pre-treatment	
No treatment	20 (34)
NSAIDs	10 (17)
PCM	18 (31)
NSAIDs + PCM	11 (19)
Adjuvant analgesic pre-treatment	
No treatment	41 (69)
Antidepressants	6 (10)
Anticonvulsants	3 (5)
Muscle relaxants	2 (3)
Others	3 (5)
Antidepressants + anticonvulsants	2 (3)
Anticonvulsants + muscle relaxants	2 (3)
Quantitative sensory testing	
Electrical pain threshold, mA	15 [5; 28]
Pressure pain threshold, kpA	1077 [737; 1436]
Cold pressor time, sec	103 [37; 120]
Cold pressor pain, NRS 0–10	8 [5; 10]
Conditioned pain modulation, kpA	80 [-5; 211]
Conditioned pain modulation, relative values	7 [-1; 21]
Situational pain catastrophizing	27 [11; 36]
Prescribed opioid treatment	
Tramadol	24 (41)
Methadone	3 (5)
Oxycodone	4 (7)
Morphine	1 (2)
Buprenorphine	24 (41)
Tapentadol	3 (5)
Morphine milligram equivalents per day (mg/day)	10 [10; 10]

Values are presented as numbers (%), means ± SD or medians [interquartile range], as appropriate.

BMI: Body Mass Index; CNS: Central Nervous System; PNS: Peripheral Nervous System; NSAIDs: Non-steroidal Anti-inflammatory Drugs; PCM: paracetamol (acetaminophen).

### Outcomes

Table 2 shows changes in primary and secondary outcomes after 14-days of opioid treatment. Treatment was clinically successful in nine patients (16%) where average pain intensity was reduced by at least 30%; 12 patients (21%) demonstrated a clinically relevant change (score ≥10 from baseline) in quality of life assessed by QLQ-C30. Eleven patients (19%) stopped opioid treatment prematurely. The most frequently reported side effect was sedation (74%) followed by constipation (49%), nausea (46%), and dry mouth (44%).

**Table 2:** Changes in primary and secondary outcomes after 14-days of opioid treatment.

Outcome	Pretreatment score (95% CI)	14-days change (post-pre) (95% CI)	P value
BPI			
Worst pain	7.2 (6.8; 7.6)	-0.3 (-0.6; 0.03)	0.12
Mild pain	4.3 (3.8; 4.8)	0.2 (-0.3; 0.6)	0.47
Average pain	6.1 (5.7; 6.5)	-0.3 (-0.7; 0.05)	0.11
Current (now) pain	5.7 (5.2; 6.3)	-0.2 (-0.8; 0.3)	0.60
Mean severity score	5.8 (5.4; 6.2)	-0.2 (-0.5; 0.2)	0.71
Mean interference score	5.9 (5.4; 6.4)	-0.7 (-1.2; -0.2)	0.02
QLQ-C30			
Global health status	55.6 (50.6; 60.6)	2.9 (-1.9; 7.7)	0.39
Physical functioning	49.7 (45.0; 54.4)	2.1 (-1.4; 5.7)	0.13
Role functioning	37.5 (30.3; 44.6)	-0.3 (-6.8; 6.2)	0.80
Emotional functioning	56.9 (50.0; 63.9)	8.9 (-4.9; 12.8)	<0.0001
Cognitive functioning	51.4 (43.2; 59.7)	4.6 (-0.3; 9.5)	0.08
Social functioning	51.4 (43.5; 59.4)	8.6 (-2.7; -14.4)	0.007
Fatigue	65.4 (59.4; 71.4)	-5.2 (-10.4; 0.1)	0.05
Nausea and vomiting	9.3 (4.8; 13.8)	5.9 (-0.3; -11.6)	0.15
Pain	79.4 (74.5; 84.3)	-6.8 (-12.3; -1.3)	0.03
Dyspnea	14.9 (9.0; 20.9)	2.9 (-3.1; 8.9)	0.57
Insomnia	61.5 (52.8; 70.2)	-10.3 (-17.2; -3.5)	0.009
Appetite loss	20.3 (12.8; 27.9)	-2.8 (-9.1; 3.5)	0.31
Constipation	14.4 (8.4; 20.3)	8.0 (1.0; 15.1)	0.05
Diarrhea	10.5 (5.2; 15.9)	-4.1 (-9.1; 0.9)	0.06
Financial difficulties	43.0 (32.7; 53.4)	-6.1 (-13.0; 0.9)	0.03

Changes in primary and secondary outcomes with 95% confidence intervals (95% CI) after 14-days of opioid treatment.

95% CI: 95% Confidence Interval; BPI: Brief Pain Inventory; Mean severity score: A composite of the four BPI pain items; Mean interference score: A composite of the seven BPI pain interference items; QLQ-C30: European Organization for Research and Treatment of Cancer Quality of Life questionnaire, each QLQ-C30 scale score ranges from 0–100. A higher score for global health status/quality of life indicates a high quality of life; a higher score for functional scales indicates a healthy level of functioning; and a high score on the symptom scale and on single items indicates a high level of symptoms or problems.

### Predictors of clinically successful treatment

Mixed-effects logistic regression output are shown in Table 3. These analyses indicated that clinically successful treatment in terms of average pain reduction to opioid treatment was associated with cold pain intensity (OR: 0.69; 95% CI: 0.53 to 0.90; *P* = 0.01), pain catastrophizing (OR: 0.82; 95% CI: 0.68 to 0.98; *P* = 0.03), relative delta (OR: 0.76; 95% CI: 0.59 to 0.97; *P* = 0.03) and beta EEG activity (OR: 1.18; 95% CI: 1.01 to 1.37; *P* = 0.04) induced by experimental cold pain. In contrast, none of the study variables were predictive of improvement in quality of life (Table 3).

**Table 3:** Associations of pre-treatment predictive factors with measures of clinically successful treatment.

	Model 1 Unadjusted				Model 2 Adjusted			
Outcome/Predictors	OR	SE	P value	95% CI	OR	SE	P value	95% CI
Δ BPI Average pain ≥30%								
EPT	1.03	0.03	0.33	0.97–1.10	1.03	0.03	0.39	0.97–1.10
PPT	1.00	0.001	0.41	0.99–1.01	1.00	0.001	0.66	0.99–1.01
CPT time	1.00	0.01	0.38	0.99–1.03	1.00	0.01	0.56	0.98–1.03
CPT pain	0.69	0.1	**0.01**	0.52–0.92	0.69	0.09	**0.01**	0.53–0.90
CPM%	0.99	0.02	0.74	0.97–1.02	0.99	0.02	0.62	0.96–1.02
S-PCS	0.85	0.05	**0.01**	0.75–0.96	0.82	0.09	**0.03**	0.68–0.98
EEG delta activity	0.80	0.08	**0.02**	0.67–0.97	0.76	0.10	**0.03**	0.59–0.97
EEG theta activity	0.87	0.19	0.54	0.56–1.35	0.92	0.20	0.70	0.59–1.43
EEG alpha activity	1.01	0.12	0.44	0.88–1.33	1.28	0.21	0.14	0.93–1.77
EEG beta activity	1.18	0.09	**0.03**	1.02–1.37	1.18	0.09	**0.04**	1.01–1.37
Δ QLQ-C30 Global health status ≥10								
EPT	1.01	0.03	0.62	0.96–1.06	1.01	0.03	0.97	0.97–1.07
PPT	1.00	0.001	0.53	0.99–1.01	1.00	0.001	0.34	0.99–1.01
CPT time	1.00	0.01	0.56	0.99–1.01	1.00	0.01	0.68	0.98–1.01
CPT pain	0.86	0.09	0.18	0.70–1.07	0.87	0.09	0.20	0.70–1.08
CPM%	1.01	0.01	0.40	0.99–1.03	1.01	0.01	0.40	0.99–1.03
S-PCS	1.00	0.02	0.90	0.96–1.05	1.01	0.03	0.77	0.96–1.06
EEG delta activity	0.95	0.06	0.38	0.84–1.07	0.89	0.07	0.17	0.76–1.05
EEG theta activity	0.87	0.18	0.49	0.58–1.30	0.76	0.19	0.27	0.47–1.23
EEG alpha activity	1.05	0.10	0.66	0.86–1.27	1.05	0.14	0.72	0.81–1.36
EEG beta activity	1.05	0.06	0.40	0.93–1.18	1.10	0.07	0.16	0.96–1.25

Associations of pre-treatment predictive factors with measures of clinically successful treatment (average pain intensity reduction ≥30% and global health status improvement ≥10), estimated odds ratios (OR) with 95% confidence intervals (95% CI) from multilevel mixed-effects logistic regression models.

Model 1: unadjusted mixed-effects logistic regression model including only the predictor variable of interest (as continuous variable) and a random intercept for participating center as explanatory variables; Model 2: mixed-effects logistic regression model including additional adjustment for sex (as dichotomous variable) and age (as continuous variable). Of note, concerning continuous predictors, the coefficient is the odds ratio for successful treatment per unit of measurement of difference in the predictor variable; OR: odds ratio; SE: standard error; 95% CI: 95% Confidence Intervals; Significant *P* values are marked in **bold**; BPI: Brief Pain Inventory; EPT: Electrical pain threshold (mA); CPM%: conditioned pain modulation (i.e. relative (%) difference between two test stimuli (pressure pain thresholds—PPT) before and after a conditioning stimuli (120 s cold pressor test—CPT) (last minus first); S-PCS: the Situational Pain Catastrophizing Scale administered in connection with CPT; relative delta (1–4 Hz), theta (4–8 Hz), alpha (8–12 Hz) and beta (12–32 Hz) bandwidth activities; QLQ-C30: European Organization for Research and Treatment of Cancer.

## Discussion

In this cohort study on a heterogeneous study population of patients with various chronic pain conditions and different underlying pain mechanisms, initiating use of both weak and strong opioids, we found evidence to support the hypothesis that opioid efficacy can be predicted before treatment based on patients’ responses to experimental pain. Our results suggest that patients with a relative ability to inhibit pain-related thoughts and feelings, reporting lower pain intensity ratings, or expressing specific electrical brain activity patterns during painful stimulation benefit most from opioid treatment.

There is emerging evidence from *experimental pain studies* that nociceptive characteristics can serve as predictors of treatment response [5]. Further, a few clinical studies have evaluated the role of pain processing in the prediction of clinically successful chronic pain treatment, but only in highly selected patient populations [9, 11]. Yarnitsky et al. showed that duloxetine, a serotonin-norepinephrine reuptake inhibitor (SNRI) believed to improve a malfunctioning pain modulation mechanism, was most beneficial in alleviating the pain in patients with painful diabetic neuropathy demonstrating inefficient conditioned pain modulation [9]. Olesen et al. found that patients with pain due to chronic pancreatitis with segmental hyperalgesia of the pancreatic viscerotome had a superior pregabalin response [11]. Our finding that the cold pressor pain response predicted opioid treatment response further stresses the potential of using quantitative sensory testing in routine clinical practice. *Brain function* is a promising biomarker for chronic pain and a neural signature of experimental pain has recently been defined in humans [22]. EEG recorded as evoked brain potentials has proven a viable tool for analyzing changes in cortical activity following administration of different analgesics [2, 23]. Specifically the activity in the delta band was changed in experimental and clinical studies following opioid administration [24]. In experimental studies the EEG response to experimental pain was also shown to predict the effect of morphine and the delta band was the main frequency band involved in differentiating between responders and non-responders to morphine analgesia [10]. This was also the case in the present study and thus it seems the delta band is closely connected to opioid analgesia, and thus a possible biomarker to be used in personalized clinical pain management interventions. The fact that the beta band also predicted successful opioid treatment has not been reported in previous studies. However, as relative EEG indices are used there is a chance that differences in the delta band could carry over to the beta band. Nonetheless, the beta band should be further investigated for its ability to predict pain and analgesia, as it has previously been shown to be involved in pain processing [25]. EEG might be a particularly interesting technology because it makes little demands on patients’ cognition and cooperation. Moreover, reliable EEG is now easily recorded using cheap commercial headsets, thus facilitating more widespread clinical use [26]. Typically, patients with high levels of *pain catastrophizing* rate their pain higher than non-catastrophizers. As such, catastrophizers are more likely than non-catastrophizers to continue to suffer from high levels of persistent pain in spite of treatment [27]. We aimed at investigating pain catastrophizing with reference to cold pressor pain, because more robust correlations with pain-related outcomes have been reported for situational measures of pain catastrophizing compared with dispositional measures [21, 28, 29]. The mechanism by which pain catastrophizing interferes with analgesic treatment response is unclear, but there is evidence to suggest that it may be related to a disruption in the endogenous modulation of pain [30]. Pain catastrophizing shares variance with negative affect constructs, such as anxiety and depression; other psychological tests might also have been suitable to elucidate a patient’s pain and inform pain-treatment strategies. However, we anticipated that additional questionnaires would only complicate and increase the length and difficulty of testing without necessarily producing better treatment prediction.

There are limitations to our study. The study was intentionally observational and representative of the real-world sample in order to mirror the daily clinical setting. Hence, we acknowledge, that our study group was very varied, representing many chronic pain related diseases and conditions that could all have influenced the treatment responses to opioids. Along this line, it is worth mentioning that, multiple pain mechanisms and outcome-relevant patient characteristics may be active to varying degrees in different patients within a chronic pain related disease or condition–leading to marked inter-subject variation in treatment effects [31].This variability in phenotypic presentation of different chronic pain related diseases and conditions has been found to be greater between patients than between different chronic pain related diseases and conditions [32–34], indicating that successful treatment is likely to be based at the level of the individual rather than at the level of the disease [31]. Furthermore, focusing on a single pain related disease or condition would have limited the generalizability of the study and reduced the sample size that could be achieved in the available study period. It is also worth mentioning that some patients will benefit from any type of intervention you offer. Thus we cannot exclude that multimodal treatment approaches usually involving more than one drug or even a placebo treatment would have had the same effect. Future studies might further standardize concurrent interdisciplinary treatment to clarify if our findings are specific enough for opioids. Another limitation is the lack of long-term follow-up. How this might affect prediction of the efficacy of a longer-term opioid prescription with a fixed mode of administration/dose is debatable. It is also uncertain whether our results can be generalized to all opioids. Our opioid response may seem low; however complete pain relief is unlikely and we may have set the threshold too high by defining a response as ≥30% pain relief. Based on the observed change from baseline in ratings of BPI pain interference items and QLQ-C30 functional scales, we found some evidence that opioids improved function; however, the number of reported side effects mostly supports the extensive evidence showing the possible harms of opioids. There are diseases and clinical circumstances under which improvement in physical function is a more realistic goal than reductions in pain and vice versa. Nonetheless, a single outcome measure including improvement in both pain relief and function is highly warranted.

In summary, the analgesic action of opioids remains unpredictable whereas most patients suffer from side effects. Hence, there is an unmet need for more personalized clinical interventions in pain management. Our results show that alterations in pain regulatory systems may be linked to successful chronic pain treatment. Our data also suggest that a test battery to investigate pain processing can easily be implemented in daily practice and may pave the road for improved pain treatment strategies.

## Supporting information

S1 STROBE Checklist STROBE Statement—Checklist of items that should be included in reports of cohort studies. (PDF)
