# Correction: Predictors of opioid efficacy in patients with chronic pain: A prospective multicenter observational cohort study

**DOI:** 10.1371/journal.pone.0224779

**Published:** 2019-10-30

**Authors:** Kasper Grosen, Anne E. Olesen, Mikkel Gram, Torsten Jonsson, Michael Kamp-Jensen, Trine Andresen, Christian Nielsen, Gorazd Pozlep, Mogens Pfeiffer-Jensen, Bart Morlion, Asbjørn M. Drewes

The Data Availability statement for this paper is incorrect. The correct Data Availability statement is: The anonymized minimal dataset underlying this study is available from figshare at https://doi.org/10.6084/m9.figshare.6269189.
